# Are training and transfer effects of working memory updating training modulated by achievement motivation?

**DOI:** 10.3758/s13421-017-0773-5

**Published:** 2017-11-28

**Authors:** Xin Zhao, Yiwenjie Xu, Junjun Fu, Joseph H. R. Maes

**Affiliations:** 1 Behavior Rehabilitation Training Research Institution, School of Psychology, Northwest Normal University, Lanzhou, China; 2 Donders Institute for Brain, Cognition and Behaviour, Centre for Cognition, Radboud University, PO. Box 9104, Nijmegen, 6500 HE The Netherlands

**Keywords:** Working memory updating training, Achievement motivation, Transfer effects, Students

## Abstract

Previous studies examining effects of working memory (WM) updating training revealed mixed results. One factor that might modulate training gains, and possibly also transfer of those gains to non-trained cognitive tasks, is achievement motivation. In the present Studies 1 and 2, students with either a high (HAM) or low (LAM) achievement motivation completed a 14-day visuospatial WM updating training program. In Study 2, the students also performed a set of tasks measuring other executive functions and fluid intelligence prior to and after training. In both studies, the HAM students displayed a larger training gain than the LAM students. Study 2 revealed that after training, both groups showed better performance on the near-transfer but not far-transfer tasks. Importantly, the differential training gain was not associated with better post-training performance for the HAM compared to the LAM students on any of the transfer tasks. These results are taken to support a modulatory role of achievement motivation on WM training benefits, but not on transfer of those benefits to other tasks. Possible reasons for the general improvement on the near-transfer tasks and the absence of a modulatory role of achievement motivation on transfer-task performance are discussed.

## Introduction

The ability to update information held in working memory (WM) is a core element of executive functioning (Miyake et al., 2000). Executive functions in general, and WM updating in particular, have been suggested to be linked to fluid intelligence (e.g., Friedman et al., 2006), everyday cognitive activities such as reasoning, reading, and arithmetic (e.g., Van der Sluis, De Jong, & Van der Leij, 2007), and psychological health (e.g., Levens & Gotlib, 2010). It is for this reason that the questions of whether WM updating ability can be enhanced through training, and whether the benefits of such training transfer to other cognitive domains, have attracted much attention in recent years. The majority of targeted studies found performance improvements on the specific WM updating task that was used during training. Moreover, these training improvements generally transferred to WM tasks closely related to the trained task(s), also known as nearest- or near-transfer effects (Melby-Lervåg & Hulme, 2013; Minear et al., 2016 ). However, research directed at the question of whether or not these training benefits transfer to tasks measuring different, non-trained cognitive functions and to everyday life functioning, also termed far-transfer effects, has revealed mixed results. Based on a review of the literature, some authors provide a positive answer (e.g., Au et al., 2015), whereas others argue that there is no convincing supporting evidence (e.g., Melby-Lervåg, Redick, & Hulme, 2016). Moreover, studies that claim to have found positive evidence for far-transfer effects and reviews that cite them have been criticized for containing a variety of methodological flaws. These include the inclusion of no or inappropriate control groups, the use of single tasks rather than multiple tasks to assess specific cognitive domains, and failing to convincingly demonstrate that the far-transfer benefits (and near-transfer benefits too, for that matter) are due to enhanced WM updating ability rather than to the learning of some non-WM related strategy, or enhancement of some basic process, such as overall processing speed or familiarity with the set of used stimuli (e.g., Melby-Lervåg & Hulme, 2016; Morrison & Chein, 2011; Shipstead, Hicks, & Engle, 2012).

### Potential moderator variables

It has been suggested, and also partly supported by empirical results, that the magnitude of training effects and near-, and possibly also far-, transfer effects may depend on a number of moderator variables. These include the specific WM updating task used for training, which may target WM capacity or a more sufficient use of the available WM capacity, for example through using some strategy, the duration and spacing of training, and the participants’ age and (baseline) cognitive abilities (e.g., Au et al., 2015; Karbach, & Verhaeghen, 2014; Melby-Lervåg & Hulme, 2013; Von Bastian & Oberauer, 2014; Weicker, Villringer, & Thöne-Otto, 2016; Zinke et al., 2014). A special class of potential moderators that have received relatively little attention in the framework of WM training and transfer studies are motivational variables (e.g., Jaeggi, Buschkuehl, Shah, & Jonides, 2014), which are the focus of the present study.

With respect to motivational variables, a common distinction is that between intrinsic and extrinsic types of motivation (Ryan & Deci, 2000). This distinction is based on the source that drives an activity. When intrinsically motivated, the activity is driven by the pleasurable feelings or satisfaction that are generated by carrying out the activity itself. On the other hand, an extrinsically motivated activity is performed to achieve some external goal, such as monetary reward. Previous WM training studies have addressed the issue of motivational moderators by manipulating the participants’ extrinsic motivation, intrinsic motivation, or both. For example, when comparing training outcomes across a number of studies, Jaeggi et al. (2014) noted that transfer effects might only occur in studies in which participants are not externally rewarded (i.e., paid) for task performance during training. However, this speculation was contradicted in a later review when controlling for outliers (Au et al., 2015). Katz, Jaeggi, Buschkuehl, Stegman, and Shah (2014) used a mixture of game-based motivational elements (among which were extrinsic elements) that were intended to improve the attractiveness of the training task and, in this way, task engagement. The data from this study suggested that the motivational elements either had no effect or actually impaired training and transfer performance. Explanations for the latter effect could be distraction from task performance or an undermining of intrinsic motivation.

Another class of studies are those examining a potential modulatory effect of intrinsic motivation as conceptualized within a personality-psychology framework. Specifically, individuals may differ in their so-called need for cognition (Cacioppo & Petty, 1982). This concept refers to individual differences in the tendency to engage in and enjoy thinking. Related to this, the conscientiousness factor of the Big Five personality test (Costa & McCrae, 1992) refers to the tendency to show self-discipline and to perform well on tasks. Still another related concept is “grit,” which refers to the tendency to persevere and have a passion for long-term goals (Duckworth, Peterson, Matthews, & Kelly, 2007). These personality traits have been considered to be potentially related to differences in the benefit that individuals might obtain from cognitive training in general and/or in the transfer of such training to non-trained cognitive domains. However, previous research specifically directed at assessing such a potential modulatory role for these individual motivational differences in a WM-training framework failed to reveal any significant modulatory effects (e.g., Minear et al., 2016; Sprenger et al., 2013; Thompson et al., 2013; but see Studer-Luethi, Jaeggi, Buschkuehl, & Perrig, 2012, and Jaeggi, Buschkuehl, Shah, & Jonides, 2014; the latter study revealed that full training compliance was only demonstrated by participants with a high need for cognition, but did not directly examine training effects in relation to achievement motivation and found no difference in transfer effects as a function of the score on this measure).

Possible reasons for finding no convincing modulatory effects for these motivational variables might be that the examined personality scales are not directly linked to differences in the effort put into the WM tasks and that the range of scores on these scales was not sufficiently large. For these reasons, and because of the relatively few studies performed in this field, we aimed to assess a potential modulatory role of another individual motivational variable that, arguably, might be more directly associated with effort differences, namely achievement motivation. Achievement motivation refers to the extent to which an individual enjoys performing challenging cognitive or academic tasks, which is linked to the capacity to anticipate positive or negative affects while performing such tasks (e.g., Nygård & Gjesme, 1973). Individuals with a high achievement motive base their self-regard on successfully employing and further advancing their skills (Atkinson, 1964). Especially the latter feature may be conducive to these individuals showing a strong task engagement, striving for an increasingly better performance when faced with an adaptive training protocol.

### Present studies

The present studies aimed to assess achievement motivation as a possible moderator of WM updating training and transfer effects. High achievement motivation may be expected to enhance task engagement and training gains. Moreover, also based on a WM training experiment using school children as participants, Jaeggi, Buschkuehl, Jonides, and Shah (2011) claimed that training-induced transfer effects are critically dependent on achieving training gains: the higher the training gains, the stronger the transfer effects. This seems plausible provided that training enhances those cognitive processes that are also importantly involved in performing the transfer task. However, it must be noted that, also purely for mathematical reasons, the mere observation of a correlation between training and transfer gain is not *sufficient* for concluding that larger training gains cause larger transfer effects (Tidwell, Dougherty, Chrabaszcz, Thomas, & Mendoza, 2014). Still, with this reservation in mind, as a first step, we aimed to assess whether there is a positive association between training and transfer gain at all to begin with. We hypothesised that adult individuals with a high achievement motivation (HAM individuals), because of their expected higher WM training gains, reflecting enhancement of WM updating ability, display a larger performance enhancement on related cognitive tasks than those with a low achievement motivation (LAM individuals). To these ends, we performed two studies. In Study 1, we first assessed WM updating task performance in the course of a multiple-day WM training program for HAM and LAM individuals. The purpose of Study 2 was to replicate the larger training gain for HAM compared to LAM participants observed in Study 1, and to examine whether this differential training gain also translates into better performance on transfer tasks for the former than the latter participants. Specifically, we examined transfer to other tasks measuring WM updating (near-transfer tests), and to tasks measuring response inhibition, interference control, task-switching, and fluid intelligence (far transfer tests). These frequently-used tests were chosen to cover the most important components of executive functioning (e.g., Miyake et al., 2000) in addition to more general (non-verbal) intelligence.

## Methods

### Participants – Study 1

One hundred participants from Northwest Normal University (Gansu, province China) were approached and asked to complete the Achievement Motives Scale (AMS; see below). The AMS score was used for selecting and grouping participants into a high achievement motivation group (HAM; *n* = 25, top 27 % of the AMS scores) and a low achievement motivation group (LAM; *n* = 25, bottom 27 % of the AMS scores). The data of one participant from the LAM group were excluded because of non-adherence to the training schedule. The mean AMS score of the HAM group (*M* = 19.56, *SD* = 7.26, 21 females) was significantly higher than that of the LAM group (*M* = -5.29, *SD* = 5.66, 20 females), *F*(1, 47) = 177.62, *p* < 0.001, *η*_*p*_^*2*^ = 0.79. Compared to the mean AMS scores obtained from previous studies (e.g., see Hagtvet & Zuo, 2000; Man, Nygård & Gjesme, 1994, range: 8−20), the LAM group especially showed a large deviation from the mean, implicating a particularly strong negative achievement motivation. The mean age of the students in the HAM group was 19.44 years (*SD* = 1.00) and that of the LAM students was 19.92 years (*SD* = 1.06). All participants had normal or corrected-to-normal vision and provided written informed consent. The study was approved by the local ethics committee.

### Participants − Study 2

As in Study 1, 100 students were approached to fill in the AMS, which was fully completed by 96 students. Participants were then grouped into a HAM (*n* = 26; top 27 %) and a LAM (n = 26; bottom 27 %) group, based on the AMS score. During training, one participant from the HAM group and six participants from the LAM group terminated participation prematurely, resulting in *n* = 25 (17 female) for the HAM group and *n* = 20 (18 female) for the LAM group. The mean AMS score was *M* = 21.88 (*SD* = 9.03) for the HAM group and *M* = -4.00 (*SD* = 4.72) for the LAM group. The between-group difference was significant, *F*(1, 43) = 134.31, *p* < 0.001, *η*_*p*_^*2*^ = 0.76. The mean age of the participants in the HAM and LAM groups was, respectively, 19.08 (*SD* = 1.04) and 19.65 (*SD* = 1.09) years. All further details were as in Study 1.

### Questionnaire (both studies)

A Chinese version (Ye & Hagtvet, 1992) of the Achievement Motivation Scale (Gjesme & Nygård, 1970) was used to measure achievement motivation. The scale consists of 30 items. Fifteen items cover the capacity to anticipate positive affects in achievement situations (*M*_s_ items; e.g., “I am attracted to situations that allow me to test my abilities”). The remaining 15 items assess the capacity to anticipate negative effects in such situations (*M*_f_ items; e.g., “I am afraid of failing in somewhat difficult situations when a lot depends on me”). Each item is answered on a 4-point Likert scale, ranging from 1 = completely disagree to 4 = completely agree. The final achievement motivation score was computed by subtracting the total score on the *M*_f_ items from the total score on the *M*s items. A higher score reflects higher levels of achievement motivation. The coefficient of internal consistency was close to acceptable (Cronbach’s α = 0.69).

### Training task (both studies)

In both studies, we employed an adaptive visuospatial *n*-back task for training that was based on an Eprime program developed by Jaeggi and colleagues (see http://wmp.education.uci.edu/software and Buschkuehl, Hernandez-Garcia, Jaeggi, Bernard, & Jonides, 2014) and that has been shown to result in near-transfer effects in previous research (Minear et al., 2016). On each trial, the participant had to track the position of a blue square that could be presented in one of eight positions on a computer monitor. The stimulus was presented for 500 ms, followed by a blank screen that was presented for 2,500 ms, resulting in a response window of 3,000 ms. When the position of the square on the present trial matched the square’s position *n* trials back in the sequence (hereafter called a target position), the participant had to press the letter A on a standard keyboard. The difficulty of the task was adapted on the basis of the participant’s performance by changing the magnitude of *n*. Specifically, *n* (the initial value of each training session was 2) was increased by 1 if the participant’s performance level (i.e., percentage of correct responses) within the current difficulty level was above 90 %. If the participant’s performance level dropped below 70 %, *n* was decreased by 1. In case of a performance level between 70 % and 90 %, *n* remained the same. Each training session lasted about 20 min and consisted of 15 blocks, each containing 20 + *n* stimuli (six target and 14 + *n* non-target positions). Participants received feedback on the percentage of trials with a correct response after each block. Training performance for each session was defined as the average *n*-back level reached during the session.

### Pre- and post-training tasks (Study 2 only)

#### Number 2-back task

This task measured WM updating ability using different stimuli compared to those used in the training task. A sequence of single digits consisting of the numbers 1−9 were presented consecutively in the middle of the screen. Each stimulus was presented for 500 ms, followed by a 2,500-ms blanc screen, resulting in a response window of 3,000 ms. The participant was required to press the letter F on a keyboard if the present digit matched the digit presented two trials back in the sequence. The participant had to press the letter J in case of a non-match trial. The test started with a block of 16 practice trials, which was repeated until the participant reached an accuracy level of >70 %. The actual test consisted of 84 trials, divided into two blocks of 42 trials each. By definition, the first two trials of each block were non-match trials. Across the task, 50 % of the trials were match trials. The dependent measures from this task were derived from signal detection theory (Stanislaw & Todorov, 1999) and were based on the proportion of hits (correctly respond with “F” on match trials) and false alarms (incorrectly respond with “F” to non-match trials). Using these measures, we computed d-prime (*d'*) as a measure of sensitivity to detect match trials among the non-match trials (higher value represents greater sensitivity), and *c* (criterion) as a measure of the tendency to respond with “match” (lower value represents stronger tendency). One participant from the HAM group did not respond at all during the pre-training task and the data from this participant were not included. This task was completed by the participants in approximately 15 min.

#### Number 3-back task

This WM updating test was identical to the 2-back task except that match trials were those on which the current digit was identical to the one presented three trials back in the sequence, and each block of 42 trials commenced with three non-match trials.

#### Running number memory span task

This task was used as additional measure of WM updating (Morris & Jones, 1990) but had a different structure compared to the *n*-back tasks. Specifically, a series of single digits ranging from 1 to 9 were presented consecutively in the center of the screen. The length of the sequence varied across trial types and could consist of 5, 7, 9, or 11 digits. On each trial, participants were asked to sequentially remember the final three digits. For example, if the presented digits were 7–6–3–1–4–5–8, the participants should have remembered 7–76–763–631–314–145–458. Finally, when a blank box was presented on the screen participants had to enter the last three digits presented (i.e. 4–5–8) using the keyboard. Each digit was presented for 750 ms, followed by a blank screen that was presented for a random time between 800 and 1,200 ms. The next digit was presented immediately thereafter. The participant first received a block of eight practice trials in which each serial length (trial type) appeared twice in a random order. The formal test consisted of two blocks of 12 trials each and the participant could have a pause between blocks. Each serial length was presented three times in each trial block. The participants completed this task in approximately 15 min. The dependent measure was based on the total number of points acquired during the task, with one point assigned for each correct digit correctly entered in the correct serial position, implicating a maximum score of 3 × 24 = 72 points. This number was converted to proportion correct responses.

#### Go/no-go task

A go/no-go task (e.g., Robertson, Manly, Andrade, Baddeley, & Yiend, 1997) was used to assess response inhibition. The task consisted of one or more blocks of practice trials and four blocks of 100 experimental trials each. Each block of 20 practice trials commenced with a black fixation cross presented for 1,000 ms in the center of the computer screen, followed by either the letter X or Y, which was presented for 600 ms. Thereafter, a blank screen was presented for 1,000 ms, followed by the next letter. The participant had to respond to each X by pressing the letter J and not respond upon presentation of the letter Y. The participant was instructed to perform the task as quickly and accurately as possible. The experimental phase of the task was initiated after reaching an accuracy level of > 85 %. Each trial of the following two experimental 100-trial blocks was identical to the trials in the practice phase. Each 100-trial block comprised 70 % trials with the letter X (go trials) and 30 % trials with the letter Y (no-go trials). The order of trials was random. During the next two 100-trial blocks, the participant had to press J to the letter Y (now go trial) and not respond to the letter X (now no-go trial). In these blocks, the percentage of Y and X trials was 70 % and 30 %, respectively. The dependent measures from this test were *d'* (sensitivity to differentially respond to go- and no-go trials) and *c* (tendency to make a “go” response), based on the number of correct responses on go trials (“hits”) and incorrect responses on no-go trials (“false alarms”). The participant could have a break between trial blocks and the total task lasted approximately 15 min.

#### Stroop task

The Stroop color–word interference task (MacLeod, 1991) was used to measure interference control. The participant was asked to indicate as quickly and accurately as possible the color in which Chinese characters (Hanzi) or the symbols “####” were printed by pressing either the letter F for the color red or the letter J for the color green. The Hanzi characters referred to either the word “red” or “green” and were printed in either red or green. The task contained three types of trial: congruent, incongruent, and neutral trials. On congruent trials, the Hanzi referring to the word “red” was printed in red and the character representing “green” was printed in green. On incongruent trials, the “red” Hanzi was printed in green and the “green” Hanzi was printed in red. Finally, on neutral trials, the symbols “####” were either printed in red or green. Each trial commenced with a 500-ms fixation cross, followed by a 1,000-ms blank screen. Thereafter, the colored Hanzi or set of symbols was presented for 1,500 ms, followed by a blank screen. The blank screen was presented for a variable duration between 600 and 1,000 ms and the next trial started immediately thereafter. The task started with a block of 18 practice trials, which was repeated until an accuracy of > 85 % correct trials had been reached. Thereafter the main task was presented, consisting of three blocks of 36 trials each. The participant could have a break between blocks. Each block consisted of 12 congruent, 12 incongruent, and 12 neutral trials. The order of trials was random and the test lasted about 15 min. The dependent measure was computed by subtracting the mean response time (RT) on congruent trials from the mean RT on incongruent trials. This difference score was based on trials with a correct response and RTs ≥ 150 ms. A high score on this index reflects weak interference control. The post-training task data from one participant from the HAM group were missing.

#### Task-switching test

This task was used to measure the ability to flexibly switch between tasks (e.g., Rogers & Monsell, 1995). A series of digits (i.e., from 1 to 9, except 5) printed in red or blue was presented in the center of the screen. Participants were instructed to either make a magnitude or parity judgment about each digit, depending on the digit’s color. In task A, all digits were red and participants had to judge whether the digit was larger than 5 or not (magnitude task). Specifically, they had to respond by pressing the letter A if the digit was smaller than 5, and the letter L if the digit was larger than 5. In task B, all digits were printed in blue and the participant had to judge whether the digit was odd or even (parity task). They had to respond by pressing the letter A if the digit was odd and the letter L if the digit was even. Participants first performed two single-task (task A and task B) practice blocks until they reached an accuracy level of > 75 %. They then continued with 20 experimental blocks: ten single-task blocks consisting of eight trials each, and ten mixed-task blocks each consisting of 17 trials. During each mixed-task block, the participant had to switch between tasks A and B on every second trial. The order of blocks was random with the constraint that two single and two mixed-task blocks were grouped together. A fixation cross appeared for 1,400 ms at the beginning of each trial block, followed by the target that was presented until the subject responded. The test lasted about 20 min. The dependent measure was the switching cost: the difference in median RT on switch and non-switch trials within mixed-task blocks, with a high score reflecting poor switching ability. Only trials with a correct response and RTs < 4,000 ms were used for the computation of the median RTs.

#### Raven’s Advanced Progressive Matrices Test

Raven’s Advanced Progressive Matrices test (RAPM; Raven, Court, & Raven, 1977) was used as a nonverbal test of fluid intelligence test. The test consists of 36 diagrams or designs with a part missing. The participant has to select the correct part to complete the designs from a number of options printed underneath. Participants completed 18 even-numbered problems before training and 18 odd-numbered problems during the post-training session. The participant was given a maximum of 15 min to complete each test. The dependent measure was the number of correctly solved problems (out of 18), which we converted into proportion correct responses.

### Procedure

In Study 1, the participants completed the WM updating task on each of 14 days in the same quiet laboratory. Prior to the first training session, they were told that the training could benefit personal cognitive functioning, level of intelligence, and academic achievement and that they would receive a small financial remuneration (10 RMB) upon completion of each training session. In Study 2, all participants first completed the pre-test tasks on four consecutive days. These measurements served to assess any potential differences in pre-training fluid intelligence and executive functioning in the HAM versus LAM individuals, and as baseline to assess changes in transfer task performance after the WM training. The RAPM was completed during the first day, the 2- and 3-back tasks on the second day, the go/no-go and Stroop tasks on the third day, and the running number memory span and task-switching tests on the fourth day. Thereafter, they completed the 14-day training phase, as described for Study 1. The training phase was followed by post-training sessions during which the transfer tasks were performed in the same order as described for the pre-training assessment sessions.

### Data analysis

The training data revealed the same pattern of results in both studies. Therefore, and to increase power, we pooled the training data from the two studies. The average *n*-back level reached was subjected to a Group (HAM, LAM) × Study (1, 2) × Session (1−14) analysis of variance (ANOVA). Follow-up analyses examining the effect of Group at each level of Session were based on the error terms from the overall analysis. For Study 2, we first computed Pearson correlations among the scores on the outcome measures from the first and last training session, and each of the pre- and post-training transfer tasks. These correlations were exploratory and taken as a measure of the extent to which the different tasks tap similar cognitive processes. Next, to assess group differences in improvement on each of the transfer tasks, we subjected the outcome measure(s) from each task to a Group (HAM, LAM) × Test (pre-, post-training test) ANOVA. This implies ten tests, and to decrease the chance of Type 1 errors we adopted the Benjamini–Hochberg procedure in which the false discovery rate (FDR) was set at 0.05, to determine statistical significance; effect sizes were expressed as partial eta-squared.

## Results

### Training

**Fig. 1:**
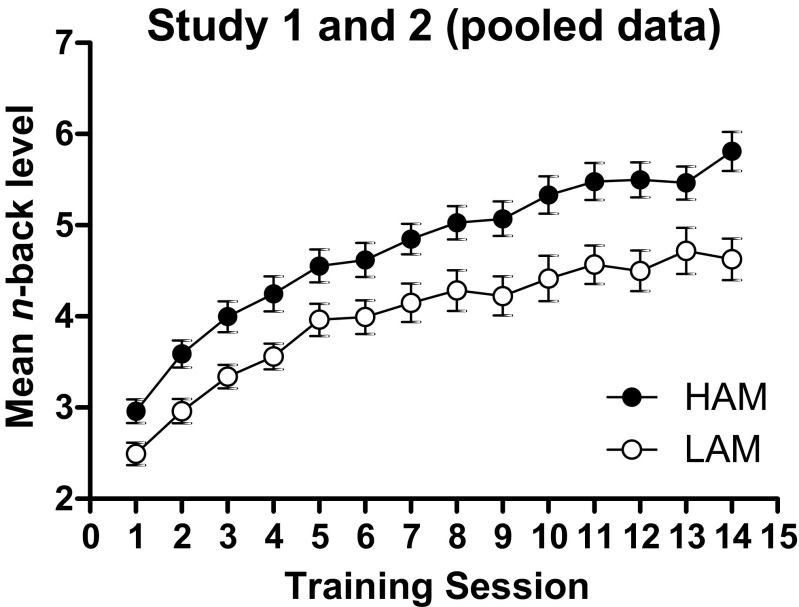
Mean (± SEM) *n*-back level reached by the group of participants with a high (HAM) and low (LAM) achievement motivation on each of the 14 working memory updating training sessions of Studies 1 and 2 (pooled)

Figure 1 displays the groups’ performance improvement across training sessions, based on the pooled data from Study 1 and 2. Both groups showed a continuous improvement as training progressed, but the improvement appeared larger for the HAM compared to the LAM group. This impression was supported by ANOVA, which revealed a Group × Session interaction, *F*(13, 1170) = 2.01, *p* = 0.02, *η*_*p*_^*2*^ = 0.02, next to a main effect of Group, *F*(1, 90) = 10.20, *p* = 0.002, *η*_*p*_^*2*^ = 0.10, and Session, *F*(13, 1170) = 124.29, *p* < 0.001, *η*_*p*_^*2*^ = 0.58 (other *p*s > 0.25). The interaction was due to the group difference being significant for each of Sessions 2−14, *F*s(1, 92) > 4.81, *p*s > 0.03, but not for Session 1, *p* = 0.08.

### Correlations among outcome measures

Table 1 presents Pearson’s correlations among the various outcome measures (for the 2-back, 3-back, and go/no-go task we limited the analysis to the most critical *d'* measure). Fluid intelligence, as measured with the RAPM, was significantly correlated with performance on most of the WM tests (especially for the post-training measurement), except for the 2-back task, and the post-training go/no-go task. Fluid intelligence was not associated with performance on the Stroop and switching tasks. Performance on the first WM training session was significantly correlated with both pre- and post-training 3-back task performance and with pre-training running memory task performance. Performance on the last WM training session was associated with post-training but not pre-training 3-back task performance, and with pre-training running memory task performance. Two-back task performance was significantly correlated with 3-back and Stroop task performance when considering the post-training tests but not the pre-training tests. Stroop task performance was significantly associated with performance on the task-switching and go/no-go tasks but this only held for the post-training measurements. Finally, in terms of test-retest correlation, the association between pre- and post-training performance was significant for the go/no-go, Stroop, switch, and Raven tasks but not for the 2-back, 3-back, and running memory tests.

**Table 1:**
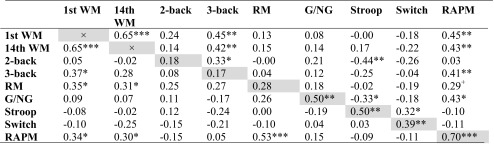
Pearson’s r between the various outcome measures

*Note*. N = 45. Values below and above the diagonal (grey cells) are based on pre-training and post-training transfer task scores, respectively. Values on diagonal represent correlation between pre- and post-training assessment. 1^st^/14^th^ WM = first and last WM training session, respectively

*2-/3-back* 2- and 3-back tasks, *RM* running memory task, *G/NG* go/no-go task, *Stroop* Stroop task, *Switch* task-switching task, *RAPM* Raven’s Advanced Progressive Matrices Test

^+^0.05 < *p* < 1; *p < 0.05; **p < 0.01; ***p < 0.001

### Transfer

Table 2 summarizes the groups’ mean performance on each of the pre- and post-training tests. ANOVAs only revealed a significant effect of the test factor for the 2-back *d'* measure, *F*(1, 42) = 31.94, *p* < 0.001, *η*_*p*_^*2*^ = 0.43, 3-back *d'* measure, *F*(1, 42) = 41.60, *p* < 0.001, *η*_*p*_^*2*^ = 0.49, 3-back *c* measure, *F*(1, 42) = 19.85, *p* < 0.001, *η*_*p*_^*2*^ = 0.32, and running memory proportion correct responses measure, *F*(1, 42) = 13.14, *p* = 0.001, *η*_*p*_^*2*^ = 0.23. The effect on *d'* and proportion correct reflected better performance on the post- compared to the pre-training test. The effect on the *c* measure reflected an overall more stringent response criterion during the post- compared to the pre-training test. All other effects were not significant.

**Table 2.** Results of ANOVA on pre- and post-training test measures for the HAM and LAM groups

Task and measure	HAM		LAM		Group *p (η*_*p*_^*2*^ *)*	Test *p (η*_*p*_^*2)*^	Group × Test *p (η*_*p*_^*2)*^
	Pre	Post	Pre	Post			
*2-back*							
- d'	2.36 (1.22)	4.30 (1.87)	2.48 (1.24)	3.98 (1.76)	0.78 (0.00)	< **0.001** (0.43)	0.47 (0.01)
- c	0.57 (0.49)	0.76 (0.80)	0.81 (0.69)	0.97 (0.76)	0.17 (0.05)	0.21 (0.04)	0.91 (0.00)
*3-back*							
- d'	2.23 (1.11)	3.41 (1.66)	1.45 (1.88)	3.52 (1.55)	0.26 (0.03)	**< 0.001** (0.49)	0.09 (0.07)
- c	0.53 (0.68)	0.75 (0.66)	0.47 (0.33)	1.23 (0.73)	0.18 (0.04)	**< 0.001** (0.32)	0.02 (0.12)
*Running memory*							
- prop. correct	0.93 (0.06)	0.95 (0.06)	0.88 (0.08)	0.94 (0.05)	0.07 (0.07)	**0.001** (0.23)	0.12 (0.06)
*Go/no-go*							
- d' - c	3.77 (0.79) -0.61 (0.57)	4.08 (0.94) -0.31 (0.47)	4.25 (1.21) -0.42 (0.62)	3.98 (1.30) -0.52 (0.60)	0.50 (0.01) 0.58 (0.01)	0.91 (0.00) 0.08 (0.07)	0.07 (0.07) 0.31 (0.02)
*Stroop*							
- interference score	54.56 (61.94)	59.26 (44.53)	64.39 (66.36)	48.51 (54.22)	0.98 (0.00)	0.52 (0.01)	0.24 (0.03)
*Task-switching*							
- response cost (ms)	234.14 (235.49)	150.74 (111.40)	205.70 (144.92)	261.20 (203.25)	0.38 (0.02)	0.63 (0.01)	0.02 (0.12)
*RAVEN*							
# correct	0.72 (0.14)	0.73 (0.13)	0.68 (0.19)	0.64 (0.23)	0.16 (0.05)	0.39 (0.02)	0.20 (0.04)

*p* values in bold are significant after correction for multiple testing using the Benjamini-Hochberg procedure. Values represent mean values (+SD)

*Pre and Post* pre- and post-training assessment

## Discussion

The present two studies revealed that WM updating training resulted in a larger training gain for individuals with a high compared to a low achievement motivation. Study 2 revealed that the differential training benefit was not associated with enhanced transfer effects for the high compared to the low achievement motivation participants. Both achievement motivation groups displayed enhanced performance on the post- relative to the pre-training tasks assessing WM but not on tasks measuring other aspects of executive functioning or fluid intelligence.

More than LAM individuals, HAM individuals base their self-regard on successfully employing and further advancing their skills (Atkinson, 1964). Moreover, compared to LAM individuals, HAM individuals are more interested in challenging tasks, and experience a more positive mood when performing such tasks when they match their skill level (Eisenberger, Jones, Stinglhamber, Shanock, & Randall, 2005). Instead, LAM individuals are prone to experience anxiety when performing challenging tasks. They tend to avoid such tasks in order to avoid failure and a corresponding negative self-evaluation (Atkinson, 1974). These differences implicate a difference in level of intrinsic motivation for performing difficult tasks and were expected to affect the effect of WM updating training. Accordingly, in terms of the present visual *n*-back training task, a higher achievement motivation should be associated with a stronger task engagement, a stronger persistence in performing the task after a mistake (e.g., see also Diener & Dweck, 1978), and a stronger motivation to reach increasing levels of *n*. The present results are in line with these predictions. Moreover, all participants were given the same training and were told that such training could perhaps have positive effects on personal cognitive functioning, level of intelligence, and academic achievement. On the assumption that just providing such information may already promote enhanced motivation to perform the training task (to the extent that the participant believes such information; e.g., see also Jaeggi et al., 2014), one could hypothesize that the LAM participants would invest as much effort in the task as the HAM participants did, with a resulting equal training gain. However, the present results suggest that this was not the case: the personality difference proved to have a stronger effect than possible positive effects from the instruction. Finally, a comparison of the scores on the AMS with other studies suggests that especially our LAM sample deviated from the average achievement motivation score in the population. This might imply that the group differences in performance on the training and transfer tasks were primarily driven by an unusually low achievement motivation by the LAM participants and less so by an excessive high achievement motivation in the HAM group.

We found no evidence that the better training performance for the HAM group was associated with better post-training performance on two tasks that had the same *n*-back task format as used during training but that incorporated a different type of stimuli, and on a task that was assumed to also tap into WM but that had a different format (the running memory task). For each of these tasks, we only found a general improvement from pre- to post-training test. In principle, there are at least two possibilities regarding the source of this effect. The first is that it merely reflected a general practise effect associated with repeated testing. However, one potential problem with this account is that such practise effect was notably limited to the WM tasks. For each of the other tasks there seemed to be room for a test-retest improvement too, which was not observed. A second possibility is that it reflected a training effect for both groups. That is, even though the LAM participants performed worse at the end of training compared to the HAM participants, the former participants still showed a training effect (improved their performance), and this training might have been sufficient to drive the improvement on the transfer WM tasks. In other words: both groups may have surpassed a critical level of training-induced improvement that was necessary and sufficient for establishing a significant near-transfer effect.

If one accepts the latter possibility, the next question would then be whether the pre- to post-training test improvements reflect a true training-induced enhanced WM updating capacity or some non-WM related process, such as the learning to use a strategy, that was also beneficial for performing the near-transfer tasks but not any of the other tasks. Evidence against the first possibility concerns the fact that not all WM task measures were significantly correlated. For example, neither pre- nor post-training 2-back task performance was significantly correlated with performance on neither the first nor last training session. Moreover, running memory task performance was associated with the first and last training session performance when considering the pre-training but not post-training assessment, and was significantly associated with neither 2- nor 3-back task performance. These findings at best suggest only partial overlap in cognitive processes involved in performing these tasks, making the hypothesis of a shared improved training-induced WM capacity as driving the general pre- to post-training improvement less likely. Further evidence against this hypothesis is the fact that no improvement was seen for RAPM performance despite the fact that a significant association was found for this measure with training-task performance, suggesting shared cognitive processes (see also, e.g., Colom, Abad, Quiroga, Shih, & Flores-Mendoza, 2008; Friedman et al., 2006).

In general, the changes that were seen in the correlations among the different outcome measures could be taken as supporting the second view, that training affected some non-WM-related process, such as the learning of a strategy. These changes, for example the fact that 2- and 3-back task performances were related after but not before training, suggest changes in the way that at least some of the tasks were approached. This suggestion is further supported by the fact that the test-retest correlations were not significant for the WM tasks, again suggesting a change in which these tasks were dealt with. Notably the test-retest correlations were significant for each of the other tasks, suggesting no difference in task approach. Arguably, the strategy learned during training (e.g., reliance on familiarity rather than active rehearsal, see also further below) was also beneficial for performing the *n*-back and running memory tasks but not the other transfer tasks. However, on this view, the facts that the correlation between training and running memory performance was stronger before than after training, and the lack of any significant correlations between running memory and 2- and 3-back task performance, are problematic. If anything, this pattern of correlations suggests no such training-induced increasingly similar task approach for the running memory task, as is assumed above to have taken place for the training and *n*-back tasks. In this respect it may be noteworthy that the running memory task in general may tap other aspects of working memory (e.g., WM span) than tasks designed to address WM updating, like *n*-back tasks (e.g., Broadway & Engle, 2010). Hence, the source of the running memory task improvement remains unclear and may perhaps partly reflect a test-retest effect (see also relatively strong test-retest correlation for this task).

Regardless of the validity of these speculations, the present results are partly in line with those reported by Studer-Luethi et al. (2012), who examined the modulatory role of conscientiousness, a concept that is related to achievement motivation. In this study, it was found that conscientiousness is positively related to performance improvement during training using a task similar to ours. However, unlike in the present study, this personality characteristic was also positively associated with improvement on near-transfer *n*-back tasks. This difference may be related to differences in the concepts of conscientiousness and achievement motivation and requires further investigation.

### Strengths, limitations, and future directions

It might be argued that, even if we would have found differential transfer effects, the design of the present experiment would not have enabled us to conclude that an enhanced post-training performance on some task for the HAM compared to LAM participants was *caused* by the former participants having benefited more from the WM training than the latter (for the moment regardless of the issue of what exactly would have been learned during training). The reason for this lies in a weakness inherent in so-called responder analyses. In a responder analysis, participants are divided into separate groups according to the gain that they display on some training program (high: “responders” vs. low: “no responders”). This between-group difference in training gain is then assessed for its association (e.g., through a correlational approach or ANOVA) with the difference in gain observed for these groups during some transfer test. As outlined by Tidwell et al. (2014), such association in itself is not informative of the direction of effects: the association could be due to gains (or even losses for that matter) occurring during either the training or transfer task and, therefore, a significant responder (group) effect does not enable one to conclude that training caused transfer task differences, or that transfer gains are modulated by benefits obtained through training. However, it is important to note that our study differed from a typical responder approach in the sense that we did not dichotomize the participants into high and low responders in an ad hoc way on the basis of the training performance data. We defined our groups prior to training and subsequently tracked their performance on the training and transfer tasks. Hence, in principle, the study could have revealed initial evidence of differential training gains differentially enhancing at least near-transfer task performance.

Another potential limitation of the present studies is the relatively small sample sizes, which might have resulted in insufficient power to detect differential transfer effects. However, a post-hoc power analysis using G*Power 3 (Faul, Erdfelder, Lang, & Buchner, 2007), with the population effect size derived from the difference in performance observed on the last training session of Study 2 between the HAM and LAM groups (*η*_*p*_^*2*^ = 0.19 = Cohen’s d = 0.48), an α level set at a level corresponding to the FDR used in the Benjamini-Hochberg correction for determining significance in the analyses of transfer-task performance (implicating a mean α level of 0.0275), and a total sample size of 45 (two groups), actually revealed a power of 0.83 to detect a significant critical Group × Test interaction in the tests for differential transfer effects.

An asset of the present studies is the use of two participant groups that only differed from one other in terms of achievement motivation while receiving identical training. Therefore, the LAM participants could be conceived of as a “control” group for estimating the magnitude of training and transfer benefits of the HAM participants. The issue of which type of control group is most adequate in the field of cognitive training is a complex matter (e.g., Green et al., 2014). Active control groups that receive some non-adaptive version of the adaptive training protocol received by the experimental group may generally be the best option, but even in this case the control and experimental groups may differ in expectation of training benefits and task demands. Use of an identical challenging, adaptive training protocol and identical instructions concerning potential training benefits in both groups avoid these confounds: differences in training and transfer gain are due to a difference in a personality characteristic related to achievement motivation rather than to different task characteristics. However, inclusion of a more traditional control group would have enabled us to directly assess test-retest improvements that are not affected by intermediate training.

A further strength of Study 2 is the use of a relatively large number of common transfer tasks that were intended to cover the most important aspects of executive functioning, specifically, WM, response inhibition, interference control, and set-shifting, in addition to fluid intelligence. However, each of these tasks may have their own drawbacks, such as being not process pure, potentially limiting their validity (e.g., Miyake & Friedman, 2012). One solution to this problem in future studies is to measure each executive function component using multiple tests, so as to enable the creation of task-non-specific latent variables (e.g., Shipstead, Redick, & Engle, 2012).

The present results can be contrasted with those reported by Zhao, Wang, Liu, and Zhou (2011). This study did reveal transfer effects of a training protocol, using a WM task that was similar to the present number running memory task, to fluid intelligence (far transfer). However, this study used children as participants, which might in general show greater plasticity than adults (see also e.g., Zhao, Chen, & Maes, 2016). Moreover, as also indicated before by the present lack of associations with most of the other WM measures, the number of running memory task may implicate different cognitive processes than *n*-back tasks do.

We interpreted the present findings as providing evidence for training affecting strategy use rather than WM updating ability. This evidence may also reflect that we did not systematically vary the number of so-called lure trials in our training task. Lure trials are trials on which the current item does not match the *n*-back item, but one of the neighboring items. Szmalec, Verbruggen, Vandierendonck, and Kemps (2011) showed that when a task does not incorporate lure trials, performance may be largely based on familiarity matching rather than on true cognitive control mechanisms such as those implied in WM updating. We did not consistently avoid the presence of lure trial, as item generation was randomized, but the percentage of coincidental lure trials was rather low (in the order of about 5 %). This may have promoted the learning of some strategy based on familiarity rather than enhancing WM updating and it remains to be investigated whether training on tasks varying in amount of lure trials also imply variations in amount of transfer to structurally different WM tasks and perhaps other, far-transfer tasks, thereby supporting evidence of genuine training-induced enhanced WM updating abilities. With respect to the changed task approach that was suggested to be mainly induced by the present training, future research should specify the nature or content of the hypothetical strategies, for example by having the participants express their thoughts during task performance. Moreover, it would be of interest to examine the effects of achievement motivation on training and transfer in children.

### Conclusions and implications

Compared to participants with a relatively low achievement motivation, students with a high achievement motivation reached larger training gains on a visuospatial WM updating task that was repeatedly presented for 14 days (Studies 1 and 2). This larger training gain was not associated with a larger improvement (from pre- to post-training test) on three near-transfer (WM) tasks: both achievement motivation groups showed an equal improvement. No improvement was found for any of the two groups on tasks assessing other aspects of executive functioning and fluid intelligence (Study 2). The present results support previous evidence of an important role of individual personality differences in modulating training benefits. These results could have implications for the use of cognitive training programs. For example, prior treatments directed at changing the individual’s experience and cognitions related to one’s own cognitive capacities in general might improve the effect of such programs. However, the present results also suggest that such improvements are limited to the trained task.
